# DNRA and Denitrification Coexist over a Broad Range of Acetate/N-NO_3_^−^ Ratios, in a Chemostat Enrichment Culture

**DOI:** 10.3389/fmicb.2016.01842

**Published:** 2016-11-24

**Authors:** Eveline M. van den Berg, Marissa Boleij, J. Gijs Kuenen, Robbert Kleerebezem, Mark C. M. van Loosdrecht

**Affiliations:** Environmental Biotechnology Group, Department of Biotechnology, Delft University of TechnologyDelft, Netherlands

**Keywords:** chemostat, denitrification, dissimilatory nitrate reduction, DNRA, Ac/N-ratio

## Abstract

Denitrification and dissimilatory nitrate reduction to ammonium (DNRA) compete for nitrate in natural and engineered environments. A known important factor in this microbial competition is the ratio of available electron donor and elector acceptor, here expressed as Ac/N ratio (acetate/nitrate-nitrogen). We studied the impact of the Ac/N ratio on the nitrate reduction pathways in chemostat enrichment cultures, grown on acetate mineral medium. Stepwise, conditions were changed from nitrate limitation to nitrate excess in the system by applying a variable Ac/N ratio in the feed. We observed a clear correlation between Ac/N ratio and DNRA activity and the DNRA population in our reactor. The DNRA bacteria dominated under nitrate limiting conditions in the reactor and were outcompeted by denitrifiers under limitation of acetate. Interestingly, in a broad range of Ac/N ratios a dual limitation of acetate and nitrate occurred with co-occurrence of DNRA bacteria and denitrifiers. To explain these observations, the system was described using a kinetic model. The model illustrates that the Ac/N effect and concomitant broad dual limitation range related to the difference in stoichiometry between both processes, as well as the differences in electron donor and acceptor affinities. Population analysis showed that the presumed DRNA-performing bacteria were the same under nitrate limitation and under dual limiting conditions, whereas the presumed denitrifying population changed under single and dual limitation conditions.

## Introduction

Denitrification and Dissimilatory Nitrate Reduction to Ammonia (DNRA) are two microbial anaerobic respiration processes that compete for nitrate and nitrite in the environment. When nitrate is reduced by denitrification, the nitrogen is released in the atmosphere as dinitrogen gas, and traces of the gaseous intermediates nitric and nitrous oxide, while DNRA will retain the nitrogen in the habitat in the form of ammonium. The different fates of the nitrogen due to these two different dissimilatory processes can have important implications (Giblin et al., 2013). For example, in wastewater treatment plants, denitrification is usually the desired process to remove the fixed nitrogen from the wastewater. DNRA can be important for nitrogen conservation in ecosystems because the ammonium-ion is generally retained in soil and sediments by absorption to the negatively charged clay minerals and therefore available for plant and microbial uptake. In contrast, the nitrate and nitrite anions are easily lost due to leaching (Silver et al., 2001).

Denitrification and DNRA can occur in similar conditions in absence of oxygen or at low oxygen concentrations (Tiedje et al., 1982; Kraft et al., 2014). For a long time, DNRA received little consideration in studies of nitrate respiration in natural and man-made ecosystems, like wastewater treatment plants. In the last decade, interest in DNRA increased since N-labeling experiments have indicated that DNRA may play a significant role in the N-cycling (Burgin and Hamilton, 2007; Kraft et al., 2011; Rütting et al., 2011; Brin et al., 2015). Although, the physiology and bioenergetics of DNRA are relatively well studied in a selected number of pure cultures, the (quantitative) role of DNRA in the environment is one of the least described of the nitrogen cycle processes (Streminska et al., 2012).

The general hypothesis is that DNRA may outcompete denitrification at low or limiting nitrate conditions and a surplus of available carbon, i.e., a high ratio of available electron donor over nitrate-nitrogen, often expressed as the molar C/N ratio (Tiedje et al., 1982; Kelso et al., 1997; Rütting et al., 2011; Kraft et al., 2014; van den Berg et al., 2015). These conditions occur for example in the rizosphere or rumen (Tiedje et al., 1982). A low C/N ratio, i.e., low or limiting available organic carbon, has been suggested to promote denitrification. This effect of the C/N ratio has been widely observed in both aquatic and terrestrial ecosystems (Burgin and Hamilton, 2007; Rütting et al., 2011; Hardison et al., 2015), but this phenomenom has rarely been reproduced in controlled mixed culture systems in the laboratory.

In batch cultures the outcome of the competition between different microorganisms is determined by the maximum specific growth rate. In continuous systems, the competition is based on affinity for the growth limiting substrate, as truly substrate limiting conditions can be applied (Gottschal, 1985). The affinity is defined as the maximum specific growth rate over the affinity constant for the limiting substrate (μ^max^/*K*) (Kuenen, 2015). This means that the competition will be won by the organism able to grow faster at certain dilution rate, at the given concentration of the growth limiting substrate (Kuenen, 2015). As nitrate limitation is an important factor for the selection of DNRA, this configuration is essential to study the nitrate reduction processes in (continuous) enrichment culture (van den Berg et al., 2015). Additionally, the use of a substrate limited continuous culture yields more reproducible and dependable data for the study of microbial competition than the batch cultivation mode (Kuenen, 2015).

Recently, two studies using lab continuous cultures addressed the effect of the C/N ratio on the end product of nitrate reduction (Kraft et al., 2014; Yoon et al., 2015). Kraft et al. (2014) obtained a marine sediment mesocosm culture performing DNRA under nitrate limitation. When switching the system to carbon limiting conditions, DNRA activity ceased and the nitrate was reduced to dinitrogen gas. In a pure culture of *Shewanella loihica* PV-4, which can catalyse both DNRA and denitrification, Yoon et al. (2015) varied the ratio of electron donor over nitrate-nitrogen by varying influent lactate and nitrate concentrations. They observed DNRA under nitrate limiting conditions and denitrificaton under carbon-limitation, and additionally observed the simultaneous occurance of both processes, when the substrate ratio was such that both lactate and nitrate were limiting. These studies described the effect of the C/N ratio in a complex microbial community and a pure culture, which was not originally isolated for its DNRA capacity. To obtain additional insight in the underlying mechanisms of the microbial competition between denitrifiers and DNRA bacteria, we studied the effect of the ratio of available electron donor over nitrate-nitrogen in a simple enrichment culture grown on acetate mineral medium in continuous culture. Acetate was chosen to avoid fermentation processes. As this ratio is different for each substrate, dependent on the electrons it can transfer, we want to specifically refer to acetate and express this ratio as Ac/N. We hypothesized that the substrate affinities for the limiting substrates will be a major parameter in the effect of the Ac/N ratio on the competition between DNRA and denitrification.

A chemostat culture was inoculated with activated sludge and fed with acetate as electron donor and nitrate as electron acceptor (van den Berg et al., 2015) at a low enough dilution rate to allow growth of both the denitrifying and DNRA bacteria. Initially, the culture was nitrate limited and performed DNRA. Then the Ac/N ratio was changed in alternating steps. The relative contribution of denitrification and DNRA in the reactor was monitored by determining the amount of nitrate-N that was converted. Steady state populations were analyzed with denaturing gradient gel electrophoresis and fluorescent in situ hybridization. A kinetic model was used to describe the system and illustrate the underlying mechanisms in the competition.

## Materials and methods

### Chemostat operation

A double-jacket glass bioreactor with an operating volume of 2 L (Applikon, Delft, The Netherlands) was used for the cultivation of the culture. The reactor broth was continuously sparged with dinitrogen gas to maintain anaerobic conditions and operated as a continuous stirred-tank reactor (CSTR). The system was inoculated with activated sludge from the wastewater treatment plant (WWTP) Leiden-Noord, The Netherlands. Before the start of the current experiments the reactor had been running continuously for a year, as described in van den Berg et al. (2015). The reactor was operated at 400 rpm with a stirrer that contained two standard geometry six-blade turbines. The reactor temperature was controlled at 20°C by means of a water jacket and a cryostat bath (Lauda, Lauda-Königshofen, Germany). The redox potential was monitored using a Redox electrode (Mettler Toledo, Tiel, The Netherlands). The pH of the reactor liquid was monitored with a pH electrode (Mettler Toledo) and was maintained at 7.1 ± 0.05 using 0.5 M HCl and 0.5 M NaOH. The pH pumps and the pH were controlled by an ADI 1030 biocontroller (Applikon). MFCS/win (Sartorius Stedim Systems, Bohemia, NY, U.S.A.) was used for data acquisition of the online measurements (redox, pH, temperature, acid dosage, base dosage).

The dilution rate of the system was controlled at 0.026 ± 0.001 h^−1^ and the influent and effluent were pumped using Masterflex® pumps. The effluent pump was controlled by a level sensor. The medium was supplied in two separate flows of a mineral medium (A) and substrate medium (B). The influent pumps, using L/S® 14 mm tubes, were set to pump 26 ml/h, thus a total of 52 ml/h influent was pumped in.

The culture media was autoclaved before use and sparged with a small flow of nitrogen gas while connected to the chemostat. Medium A contained per liter: 23.5 mmol NaNO_3_, 22.0 mmol KH_2_PO_4_, 1.2 mmol ${\text{MgSO}}_{4}^{.}$7H_2_O, 1.5 mmol NaOH, 1.5 mg yeast extract and 5 ml trace element solution (Vishniac and Santer, 1957), with the ${\text{ZnSO}}_{4}^{.}$7H_2_O concentration reduced to 2.2 g per liter. For the Ac/N ratios of 0.93 and 0.66, NH_4_Cl was added to medium A (Table 1). Medium B contained varying concentrations of NaCH_3_COO^.^3H_2_O (Table 1) in order to create the different Ac/N ratios.

**Table 1:** **The acetate concentrations in medium B, used to obtain the different Ac/N ratios for the medium supplied to the chemostat, and the ammonium concentrations in medium A, added as N-source for the cultures lacking measurable ammonium production by DNRA**.

**Days**	**Ac/N (mol/mol)**	**Acetate (mM)**	**Ammonium (mM)**
1–12	1.87	44.1	–
13–24	1.50	35.3	–
25–38	1.08	25.5	–
39–66	1.23	29.0	–
67–100	0.93	22.0	–
101–109	0.93	22.0	2.2
110–123	0.93	22.0	2.8
124–171	1.16	27.3	–
172–200	0.66	15.4	2.8

### Analytical procedures

Oxygen, carbon dioxide, nitric oxide and nitrous oxide concentrations in the headspace of the reactor were monitored in dried gas using a gas analyzer (NGA 2000, Rosemount, Chanhassen, MN, USA). The flow of nitrogen gas to the reactor was kept at 100 Nml min^−1^ using a mass flow controller (Brooks Instrument, Ede, The Netherlands), to maintain sufficient flow through the gas analyzer (80 ml min^−1^).

Samples taken from the reactor were centrifuged and supernatants were used for analysis of acetate and nitrogen compounds. The acetate concentration in the liquid phase was measured by High Performance Liquid Chromatography using an Aminex HPX-87H column (*T* = 60°C) from Bio-Rad Laboratories (Hercules, CA, USA) coupled to a UV and RI detector using phosphoric acid (0.01 M) as eluent, with a lower limit of detection of 0.1 mM. A rapid qualitative indication of the nitrite- and nitrate-concentration in the reactor was obtained with test strips (Merck Millipore, Carrigtohill, Ireland). When this was not zero, the qualitative measurements for nitrate-, nitrite- and also ammonium-concentrations were performed spectrophotometrically with commercial cuvette test kits (Hach Lange, Düsseldorf, Germany). Nitrate concentrations as low as 0.23 mgN/l (0.02 mM) could be measured with this method. Sulfide was not detectable (<0–5 μmol/l).

To determine the biomass concentration, the reactor effluent was centrifuged (10,000 rpm for 20 min) and the pellet was dried at 105°C. Subsequently the ash content was subtracted to obtain VSS concentration. The ash content was determined by burning the organic parts of the dried pellet at 550°C. Protein content of the biomass was measured using the Uptima BC Assay Protein Quantitation Kit (Interchim, Montluçon, France). The heme content of the biomass was measured in cell suspensions, with 0.7 mg/ml protein for the DNRA biomass and 1.0 mg/ml protein for the denitrifying biomass. The absorption spectra for the heme content in the cells were recorded on an Olis DW2000 (Bogart, GA, USA) double beam spectrophotometer. Solid dithionite was used as the reductant to measure the reduced spectrum.

The biomass composition was calculated from the measured Total Organic Carbon (TOC) and Total Organic Nitrogen (TON) of washed biomass pellets, using a TOC-L CPH/CPN analyzer (Shimadzu Benelux, 's-Hertogenbosch, The Netherlands). TOC was determined as Total Carbon (TC) subtracted by Inorganic Carbon (IC) (TOC = TC − IC). Biomass composition was measured for several steady states and did not significantly differ for the different populations. In our calculations we used the average of 0.23 ± 0.01 mol N per C-mol biomass.

A balance of degree of reduction and a charge balance of incoming and exiting elements in the chemostat were set up to verify the consistency of our measurements. The ammonium production was attributed to nitrate reduction by DNRA. The nitrogen not accounted for in ammonium, nitrate, nitrite or biomass was assumed to be converted to N_2_. The concentration of volatile suspended solids (VSS) was used as biomass concentration. Value comparisons were evaluated using an unpaired student *t*-test or linear regression analysis. For the computation of the CO_2_ production rate from the off gas partial pressure we used the molar gas volume 24.5 l/mol. Losses by wash out of dissolved CO_2_ and ionized species are included in the balancing.

### Microbial population analysis

The microbial community structure was analyzed by denaturing gradient gel electrophoresis (DGGE). Biomass samples were collected from the reactor and centrifuged and stored at −20°C. The genomic DNA was extracted and analyzed as described by van den Berg et al. (2015). The set of primers used is the 341F (containing a 40-bp GC clamp) and 907R (Schäfer and Muyzer, 2001). The obtained sequences were corrected using the program Chromas Lite 2.1.1 (http://technelysium.com.au) and then compared to sequences stored in GenBank using the Basic Local Alignment Search Tool (BLAST) algorithm (http://www.ncbi.nlm.nih.gov/blast). The sequences have been deposited in the GenBank under accession numbers KT317069 to KT317073, KX002073 and KX002074.

Fluorescent In Situ Hybridization (FISH) was performed as described by Johnson et al. (2009), using a hybridization buffer containing 35% (v/v) formamide. The applied probes are listed in Table 2. The general probe mixture EUB338 labeled with Cy5 was used to indicate all eubacteria species in the sample (Amann et al., 1990; Daims et al., 1999). The Beta42a probe, labeled with Cy3 (plus an unlabeled Gamma42a probe, to minimize erroneous hybridizations of Beta42a; Manz et al., 1992), was used to target the denitrifiers and a probe for Deltaproteobacteria (Delta495) labeled with FLUOS was used to target the DNRA bacteria. Probes were synthesized and 5′-labeled with either the FLUOS or with one of the sulfoindocyanine dyes Cy3 and Cy5 (Thermo Hybaid Interactiva, Ulm, Germany). Slides were observed with an epifluorescence microscope (Axioplan 2, Zeiss, Sliedrecht, The Netherlands), and images were acquired with a Zeiss MRM camera and compiled with the Zeiss microscopy image acquisition software (AxioVision version 4.7, Zeiss) and exported as TIFF format. The relative abundances of the bacteria were based on a cell count of four randomly selected subsections of each picture, counting at least 100 cells per section.

**Table 2:** **Probes used in FISH analysis of the culture**.

**Probe**	**Sequence (5′-> 3′)**	**Dye**	**Specificity**	**References**
EUB338mix	gcwgccwcccgtaggwgt	Cy5	Most bacteria	Amann et al., 1990; Daims et al., 1999
Beta42a	gccttcccacttcgttt	Cy3	Betaproteobacteria	Manz et al., 1992
Gamma42a	gccttcccacatcgttt	none	Gammaproteobacteria	Manz et al., 1992
Delta495	agttagccggtgcttcct	Fluos	Deltaproteobacteria	Loy et al., 2002

### Model description

A computational model was developed to describe the competition between nitrate reduction to dinitrogen gas (denitrification, DN) and nitrate reduction to ammonium (ammonification, AM) in a chemostat. The model was based on Monod-kinetics with potentially two limiting substrates, nitrate (*NO3*) and/or acetate (*AC*). The actual growth rate of the microorganisms catalyzing both conversions was consequently described as shown in equation 1.

(1) $$\begin{array}{c} \mu =\mu^{max}\cdot \frac{NO3}{NO3\;+\;K_{NO3}}\cdot \frac{AC}{AC\;+\;K_{AC}} \end{array}$$

In this equation *NO3* and *AC* are the concentrations for nitrate and acetate respectively, μ^*max*^ is the maximum specific growth rate (*molX*·*molX*^−1^·*h*^−1^) and *K*_*NO3*_ and *K*_*AC*_ (*mM*) are the affinity constants for nitrate and acetate respectively. To describe process stoichiometries in the kinetic model, the overall growth reactions for denitrification and DNRA obtained from measurements were used (equations 2, 3). Thus, implemented is nitrate reduction to the pathway end-products, despite the branching of the nitrate reduction pathways at nitrite. The kinetic parameter values used are presented in **Table 7**.

The resulting system was numerically solved using the steady state assumption μ = *D* where *D* equals the dilution rate (*L*·*L*^−1^·*h*^−1^) using a two-step approach. First the effluent concentrations acetate and nitrate were calculated assuming that only denitrification or ammonification occurred at Ac/N ratios in the feed ranging from 0.6 to 2.0. If the steady state effluent concentrations of acetate and nitrate were both lower for denitrification, this process will outcompete the ammonification process, and vice versa. If this is not the case ammonification and denitrification will coexist and there is a unique solution for the effluent acetate and nitrate concentrations where both the growth rates of denitrification and ammonification are equal to the dilution rate. The full model is made available in the supplementary materials.

## Results

### Reactor operation

The influence of the mass ratio of acetate and nitrate (Ac/N ratio) on the competition between denitrification and DNRA was analyzed using an anaerobic enrichment culture. Nitrate was used as electron acceptor and N-source and acetate as electron donor and carbon source. Variable Ac/N ratios were obtained by varying the acetate concentration in the influent. Ac/N-ratios were alternated non-linearly in time (Table 1) to avoid gradual adaptation. The culture was assumed to be in steady state when the conversions observed were constant for at least five volume changes.

Since nitrogen fixation is unlikely to occur in presence of ammonium, the ammonium production was attributed to nitrate reduction by DNRA. In steady state nitrite was not detected, and in the off-gas no nitric oxide or nitrous oxide could be detected (both detection limits of 5 ppm). Although, 0.65 mM of sulfate was present in the influent, sulfate reduction was considered negligible, because no sulfide was detected (detection limit 0.5 μmol/l).

**Figure 1:**
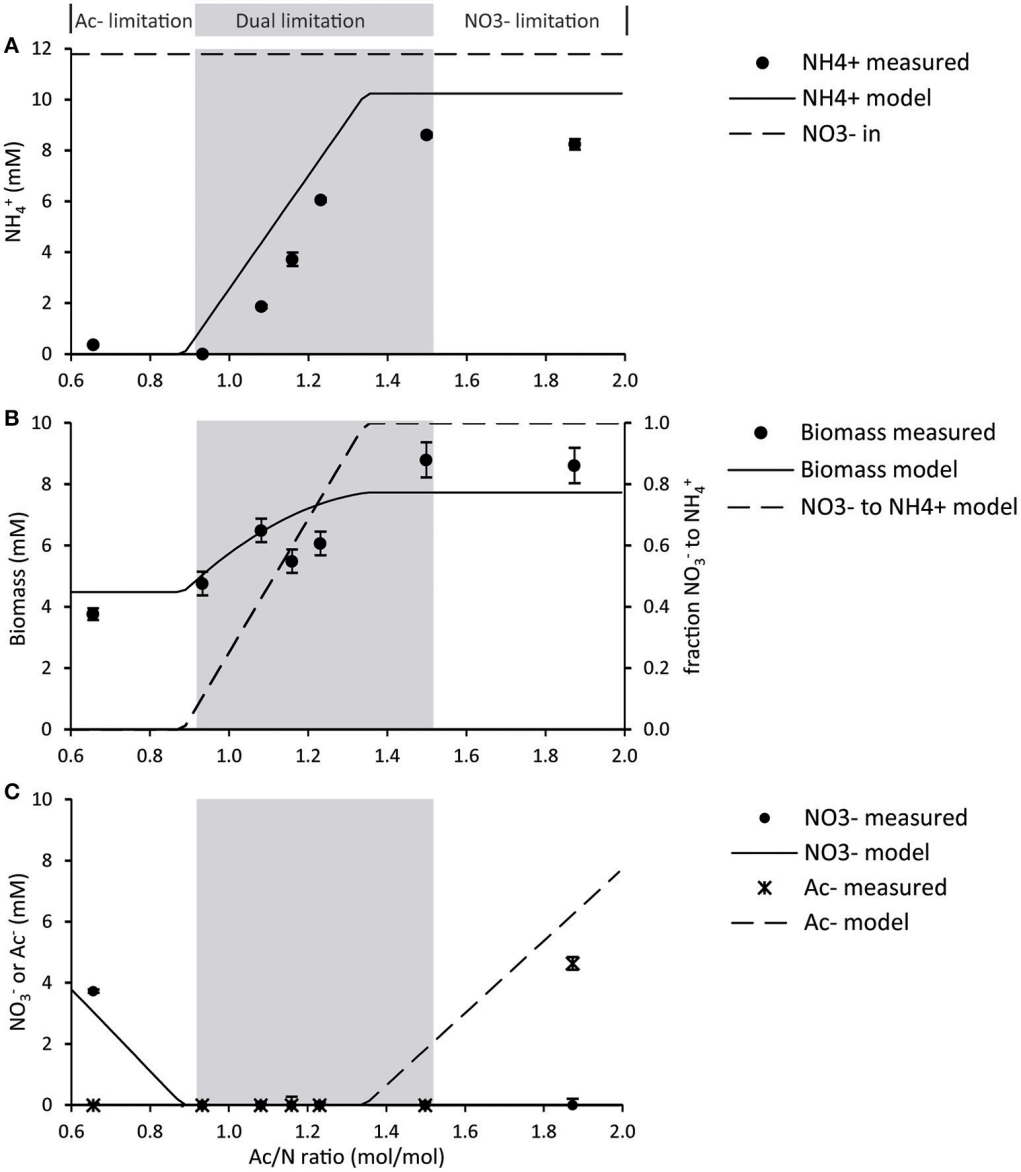
**Steady state reactor concentrations were measured for each cycle**. Average deviations were obtained from daily measurements during the steady state (*n* > 3). **(A)** Ammonium concentrations measured and modeled for different Ac/N ratios. As a reference, the influent nitrate concentration is shown. **(B)** Biomass concentrations measured and modeled. The modeled fraction of DNRA biomass is shown for reference. **(C)** Nitrate and acetate concentrations, measured and modeled.

The initial culture was enriched and grown at a high Ac/N ratio of 1.87 mol/mol in the influent (Figure 1A). This resulted in nitrate limitation in the reactor, while acetate was in excess: 15 ± 2 % of the nitrate was assimilated and 70 ± 3% was reduced to ammonium via DNRA. The remaining 15% of nitrate was assumed to be reduced to dinitrogen gas, and thus denitrified. However, this remains to be verified. At this high Ac/N ratio, the biomass yields were 18.0 ± 1.1 g VSS/mole nitrate and 12.3 ± 1.6 g VSS/mole acetate (0.62 ± 0.04 mg protein/mg VSS) and the C/N content of the biomass was 0.22 ± 0.1 molN/molC. The redox potential in the reactor under these conditions was −450 mV and the color of the mixed culture was pink/reddish, due to high heme content of the biomass (redox spectra in Figure S1). These observations showed a good reproducibility of the previous enrichment in the same conditions (van den Berg et al., 2015).

A low Ac/N ratio of 0.66 resulted in acetate limiting conditions in the reactor (Figure 1A), with excess of nitrate. Under these conditions, no ammonium was measured, hence DNRA activity was undetectable. Here, ammonium was added to the medium for growth. The biomass yield of this culture was 11.6 ± 1.4 g VSS/per mole nitrate, which is lower than the yield of the culture dominated by the DNRA bacteria (*p* = 0.0003). The yield on acetate was comparable for both denitrification and DNRA (*p* = 0.1288) (Table 3), as well as the C/N content and protein content of the biomass. The redox potential in the reactor at denitrifying conditions was −160 mV and the color of the broth was yellowish, as the enrichment culture did not have a high heme content like the DNRA culture (redox spectra in Figure S1).

**Table 3:** **Biomass yield on acetate and nitrate for the different COD/N ratios**.

**Ac/N (mol/mol)**	**Biomass yield**	
	**g VSS/mol acetate**	**g VSS/mol nitrate**
0.66	11.3 ± 1.8	11.6 ± 1.4
0.93	10.7 ± 1.1	9.9 ± 0.8
1.08	12.5 ± 1.2	13.6 ± 0.9
1.16	9.9 ± 1.1	11.5 ± 0.9
1.23	10.3 ± 1.2	12.7 ± 1.1
1.50	12.3 ± 1.4	18.4 ± 1.6
1.87	12.3 ± 1.6	18.0 ± 1.1

*Yields and average deviations were calculated using the average volatile suspended solids of steady state measurements and the average amount of substrate that was consumed*.

**Figure 2:**
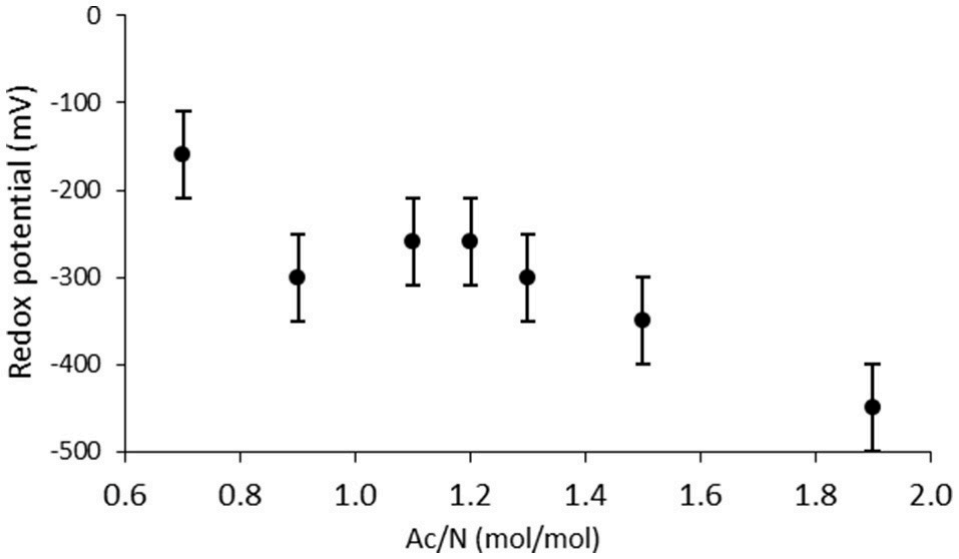
**Redox potential measured in the culture at different Ac/N ratios**. A linear correlation could be observed.

At Ac/N ratios between 0.93 and 1.50, a dual limitation of both acetate and nitrate was observed, as residual concentrations of both acetate and nitrate were not detected. The ammonium production decreased with the decreasing Ac/N ratios (*p* = 0.0044; *R*^2^ = 0.9528), indicating that DNRA became less dominant (Figure 1A). Other observations confirming a decrease in DNRA activity were the decrease in acid consumption and the change in color of the culture, which became less red and more yellow as the Ac/N ratio was decreased. Furthermore, the biomass yield on nitrate decreased with the diminishing of DNRA activity in the reactor (*p* = 0.0327, *R*^2^ = 0.8255), while the biomass yield on acetate did not change significantly (*p* = 0.5883, *R*^2^ = 0.1085) (Table 3). Hence, as acetate conversion decreased with Ac/N ratios, the biomass concentration decreased as well (Figure 1B). An overview of all steady state conversion rates is shown in Table 4. The redox potential in the reactor for the steady states with both substrate limiting conditions ranged from −260 ± 50 to −350 ± 50 mV (Figure 2). Although, the redox values are somewhat unstable, a trend in the redox potential could be observed.

**Table 4:** **Steady state conversion rates and balances for the enrichment cultures at different Ac/N ratios**.

**Ac/N**	**Compound conversion rates (mmol.h**^**−1**^**)**					**Balance residuals (%)**	
**(mol/mol)**	**HAc**	**${\text{NO}}_{3}^{-}$**	**H^+^**	**CH_1.8_O_0.5_N_0.2_**	**${\text{NH}}_{4}^{+}$**	**Reduction**	**Charge**
0.66	−0.39 ± 0.01	−0.44 ± 0.08	−0.78 ± 0.04	0.19 ± 0.01	0.00 ± 0.01	1%	4%
0.93	−0.55 ± 0.02	−0.59 ± 0.01	−1.07 ± 0.02	0.24 ± 0.02	0.00 ± 0.01	7%	6%
1.08	−0.64 ± 0.02	−0.59 ± 0.01	−1.26 ± 0.02	0.32 ± 0.02	0.09 ± 0.01	6%	5%
1.16	−0.55 ± 0.03	−0.59 ± 0.01	−1.45 ± 0.04	0.27 ± 0.02	0.19 ± 0.01	12%	0%
1.23	−0.72 ± 0.02	−0.59 ± 0.01	−1.50 ± 0.04	0.30 ± 0.02	0.30 ± 0.01	9%	8%
1.50	−0.88 ± 0.02	−0.59 ± 0.01	−1.75 ± 0.03	0.42 ± 0.03	0.43 ± 0.01	10%	9%
1.87	−0.86 ± 0.05	−0.59 ± 0.01	−1.87 ± 0.08	0.43 ± 0.03	0.41 ± 0.01	9%	1%

*The conversion rates were calculated using the measured concentrations of the compounds. Standard deviations were obtained from daily measurements during the steady state (n = 3–4 for biomass, n = 4–5 for other compounds). When substrates were completely consumed standard deviations were estimated from concentration variations due to inaccuracy of medium preparation. The nitrogen and carbon balance are unavailable since N_2_ and CO_2_ were not measured*.

### Microbial community structure

**Figure 3:**
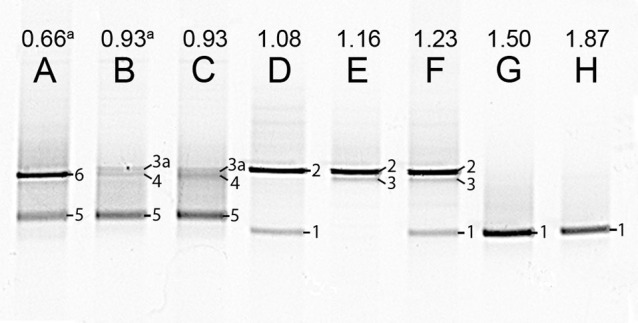
**Photograph of DGGE gel with the PCR products of the 16S rRNA genes from steady state reactor samples, with different Ac/N ratios (cropped; no other bands were present in the lanes)**. The numbers on the right side of the bands correspond to the markers in Table 5. Bands labeled with the same number, contained the same sequence. Note that for the culture in lane A denitrification is dominant and in H DNRA is. The unprocessed DGGE photo is included in Figure S2. (a) The influent was supplemented with 1.4 mM ammonium.

**Figure 4:**
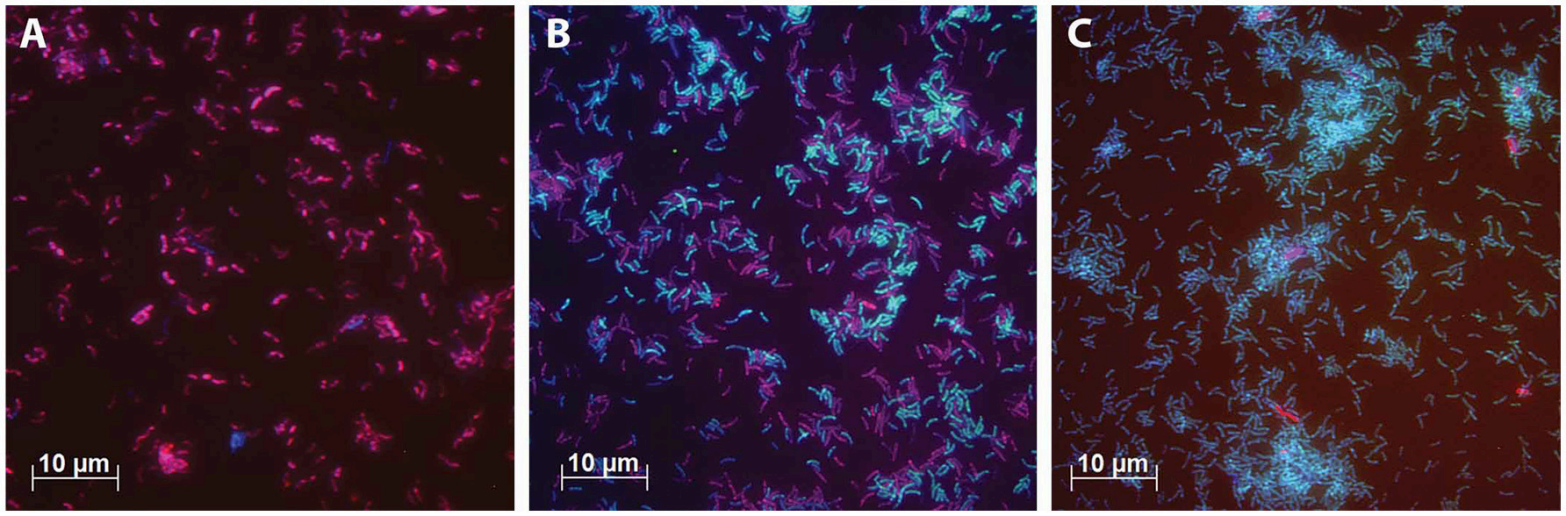
**FISH microscopic photographs of steady state cultures**. The cells were stained with Cy5-labeled probes for bacteria (EUB338mix, blue), Cy3-labeled probes for *Betaproteobacteria* (Beta42a, red) and FLUOS-labeled probes for *Deltaproteobacteria* (Delta495, green). Cells that are purple indicate cells to which the probes EUB338mix and Beta42a were hybridized. Cells that are light blue indicate cells to which the probes EUB338mix and Delta495 were hybridized. **(A)** The culture grown with Ac/N ratio of 0.66. **(B)** The culture grown with Ac/N ratio of 1.08. **(C)** The culture grown with Ac/N ratio of 1.87.

The microbial populations in the reactor steady states were analyzed by DGGE and verified by FISH (Figures 3,4, respectively). DGGE bands were sequenced and analyzed using BLASTn (Table 5). For the DNRA dominated community as obtained in nitrate limiting conditions, one dominant genotype was observed on the gel (lane A, Figure 3), which is most closely related to *Geobacter luticola*. This is the genotype corresponding to the organism performing DNRA, as is explained in the discussion. When both nitrate and acetate were limiting and denitrification and DNRA coexisted, two other dominant genotypes appeared (lane C–E, Figure 3). One of those genotypes is most closely related to *Geobacter lovleyi* (band 3, Figure 3), and 100% similar to the DRNA genotype found in our previous study (van den Berg et al., 2015). Thus, this *Geobacter* genotype is assumed to be responsible for DNRA, just like the *G. luticola* related organism. Alignment of the sequences of the bands 1 and 3 showed 97% similarity. The other genotype that appeared when both nitrate and acetate were limiting (Ac/N 1.08–1.23 mol/mol) related to *Azospira oryzae* (band 2, Figure 3), which was most likely responsible for the denitrification. When the Ac/N ratio was 0.93 (lane F, Figure 3), a genotype related with 96% identity to *Geobacter lovleyi* (band 3a, Figure 3) remained, with 98% sequence similarity to genotype 3. The denitrifier with the *A. oryzae* genotype had disappeared and two other dominating genotypes were found, next to the *Geobacter* genotype. These genotypes (band 4 and 5, Figure 3) were highly similar, 98% similarity, and for both the closest cultivated relative was *Sulfurisoma sediminicola*. Under acetate limiting conditions (Ac/N 0.66, lane H, Figure 3), two genotypes dominated. The *Geobacter* genotype had disappeared and one of the *Sulfurisoma sediminicola* genotypes remained. A new genotype appeared (band 6, Figure 3), which was most closely related to *Variovorax boronicumulans*.

**Table 5:** **BLASTn results' best alignments for the different band sequences (Figure 3)**.

**Band**	**Description**	**Class**	**Identity (%)**	**Accession number**
1	*Geobacter luticola*	*Deltaproteobacteria*	97	AB682759.1
2	*Azospira oryzae* strain N1	*Betaproteobacteria*	100	DQ863512.1
3	*Geobacter lovleyi* strain SZ	*Deltaproteobacteria*	97	NR_074979.1
3a	*Geobacter lovleyi* strain SZ	*Deltaproteobacteria*	96	NR_074979.1
4	*Sulfurisoma sediminicola*	*Betaproteobacteria*	94	AB842427.1
5	*Sulfurisoma sediminicola*	*Betaproteobacteria*	97	AB842427.1
6	*Variovorax boronicumulans*	*Betaproteobacteria*	99	JQ692103.1

Because there was no DNRA performed at low Ac/N ratios (0.93 and 0.66 mol/mol), the ammonium concentration was below the detection limit in the reactor, and nitrate was also used for assimilation. To investigate if nitrate or ammonium availability affected the denitrifying community at low Ac/N ratios, the medium was supplemented with 1.4 mM ammonium for 20 volume changes. This was sufficient for growth, and an excess of 0.2 mM residual ammonium remained in the reactor. Supplementing the medium with ammonium resulted in no identifiable differences in functional performance of the system (biomass *p* = 0.5329) and in the DGGE analysis result (compare lane F and G, Figure 3). So the type of N-source for assimilation did not change the denitrifying population.

As artifacts occur in DGGE analysis (Neilson et al., 2013), FISH analysis was performed to confirm these results. The DNRA performing bacteria found with DGGE analysis belong to the class of *Deltaproteobacteria* and the bacteria identified in the denitrifying cultures all belong to the *Betaproteobacteria* (Table 5). Therefore the relative abundance of denitrifying and DNRA performing bacteria can be seen with FISH using probes for *Beta*- and *Deltaproteobacteria* respectively. For each steady state, the relative abundance of these bacteria was estimated (Table 6) to illustrate its correspondence with the trend observed with the culture conversions and DGGE result. Figure 4 shows FISH photos of three steady state populations. The culture grown with the highest Ac/N ratio consists of almost only *Deltaproteobacteria*. With the decrease of the Ac/N ratio, the relative abundance of the *Deltaproteobacteria* decreases and the relative abundance of the *Betaproteobacteria* increases (*p* <0.0001, *R*^2^ = 0.9980). The cultures grown with the lowest Ac/N ratio show almost only *Betaproteobacteria*.

**Table 6:** **Relative abundances of the *Betaproteobacteria* and *Deltaproteobacteria* in the steady state populations obtained from cell counts of the FISH analyses**.

**Ac/N (mol/mol)**	***Betaproteobacteria* (%)**	***Deltaproteobacteria* (%)**
0.66	96 ± 2	0 ± 0
0.93	85 ± 1	15 ± 1
1.08	46 ± 3	54 ± 3
1.16	38 ± 5	62 ± 5
1.23	25 ± 1	75 ± 1
1.50	1 ± 1	99 ± 1
1.87	1 ± 1	99 ± 1

*The indicated average deviations relate to the cell counts and not to accuracy of the FISH analysis*.

### Modeling the results

A mathematical model was developed to describe the experimental results obtained and to clarify co-occurrence of denitrification and ammonification at the intermediate Ac/N ratios investigated. As the model describes the overall growth reactions, branching of the nitrate reduction pathways at nitrite was not incorporated. To validate the model structure proposed in the material and methods section, the stoichiometry of both processes needs to be identified first.

Given that at low Ac/N ratios denitrification was found to dominate the process, the stoichiometry of the denitrification (equation 2) was calculated from the measured biomass yield on acetate (−0.49 Cmol/mol).

(2) $$\begin{array}{l} -2.1\cdot C_{2}H_{3}O_{2}^{-1}\;-\;2.3\cdot \;NO_{3}^{-1}\;-\;4.4\cdot H^{+1}\;+\;\;1\;\cdot \;CH_{1.8}O_{0.5}N_{0.2}\\ \;+1.1\cdot N_{2}\;+\;3.1\;\cdot \;CO_{2}\;+\;\;4.4\cdot \;H_{2}O \end{array}$$

At high Ac/N ratios ammonification was strongly dominant and the reaction stoichiometry for nitrate ammonification (equation 3) was derived from the biomass yield on acetate, which was on average found to be comparable to denitrification (−0.49 Cmol/mol).

(3) $$\begin{array}{l} -2.1\cdot \;C_{2}H_{3}O_{2}^{-1}-\;1.5\;\cdot \;NO_{3}^{-1}\;-\;4.9\cdot H^{+1}\;\;+1\;\;\cdot \;CH_{1.8}O_{0.5}N_{0.2}\\ +\;1.3\cdot NH_{4}^{+1}\;+\;3.1\cdot CO_{2}\;+\;2\cdot H_{2}O \end{array}$$

The maximum specific growth rate value for the ammonifying culture was estimated from exponential growth curves measured during transition from low to high Ac/N-ratios (data not shown). The affinity constant for nitrate of the DNRA bacterium was estimated from reactor nitrate concentrations that were measured under nitrate limiting conditions (data not shown). When acetate limitation was observed, residual acetate concentrations were at all times below the detection limit of the used methods. The values for the affinity constants of the denitrifying community were therefore obtained from literature (Gujer et al., 1999; Scholten et al., 2002). An overview of the kinetic parameter values is presented in Table 7.

**Table 7:** **Parameter values used for modeling denitrification and ammonification in a chemostat culture**.

**Parameter**	**Symbol**	**Unit**	**DNRA**	**DN**
Maximum specific growth rate	μ^*max*^	*h*^−1^	0.052	0.086
Affinity constant for nitrate	*K*_*NO*3_	μ*M*	2	10
Affinity constant for acetate	*K*_*AC*_	μ*M*	10	10
Affinity for nitrate	μ*^max^*/*K*_*NO*3_	*h*^−1^·μ*M*^−1^	26.2	8.6
Affinity for acetate	μ*^max^*/*K_AC_*	*h*^−1^·μ*M*^−1^	5.2	8.6

*The origin of the different values in the table is explained in the text*.

Analysis of the affinity of both processes for nitrate and acetate in a chemostat, as identified by the value for μ^max^/*K* in Table 7, shows that the model correctly describes the dominance of denitrification in acetate limiting conditions (Ac/N ratios smaller than 0.93), and that of DNRA at nitrate limiting concentrations (Ac/N ratios higher than 1.50). It should be noted that although, the affinity constants in the model were roughly estimated from literature and preliminary experimental data, the model output in terms of the ratios of denitrification versus DNRA is largely independent of the absolute affinity constant values. The ratio of the affinity constants is the main factor determining the relative contribution of DNRA or denitrification in the conversions in the chemostat.

Also at the intermediate Ac/N ratios the model adequately describes the co-occurrence of denitrification and ammonification (Figure 1A). Total biomass concentrations as predicted from combined denitrification and ammonification correspond well to the measured biomass concentration (Figure 1B). Effluent ammonium concentrations due to DNRA are always overestimated by approximately 15% by the model due to partial reduction of nitrate to dinitrogen gas in our experiments as described previously.

## Discussion

### Chemostat system

In this study the influence of the Ac/N ratio on the competition for nitrate between denitrification and DNRA was investigated in an open continuous culture enrichment system. We used acetate as the single non-fermentable substrate. We observed in this system that within a remarkably wide range of Ac/N ratios dual substrate limitation and co-occurrence of both DNRA and denitrification occurred.

To describe the basic behavior of our system, we made a kinetic model to describe substrate competition and co-occurrence of DNRA and denitrifiers, omitting nitrate reduction to nitrite as a possibility. As shown in Figure 1, the model correctly describes the experimentally observed co-occurrence of denitrification and DNRA at intermediate Ac/N ratios.

In the chemostat steady states with one limiting substrate we observed a domination of one of the two different nitrate respiration processes. The DNRA bacteria dominated during nitrate limitation, indicating they have a higher affinity for nitrate than the denitrifying bacteria. The denitrifiers dominated under acetate limiting conditions, indicating a higher affinity for acetate for the denitrifiers than for the DNRA bacteria.

The biomass yield of the DNRA culture was higher than the yield of the denitrification culture per mole nitrate, whereas these yields were similar per mole of acetate. A comparison of the DNRA and denitrification yields found in this system, with theoretically expected and other empirical yields can be found in the discussion of van den Berg et al. (2015).

**Figure 5:**
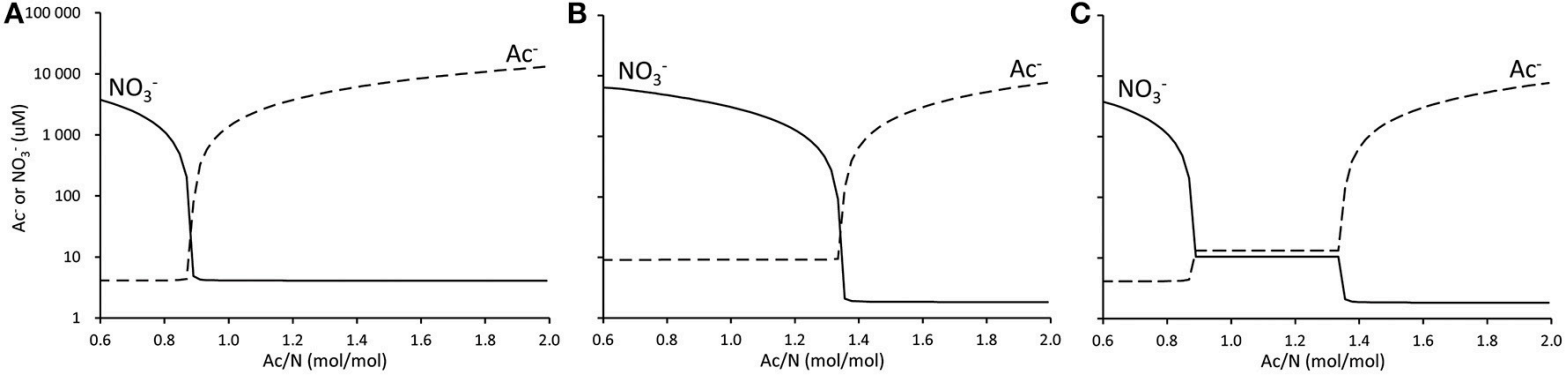
**Calculated change in residual nitrate (solid line) and acetate (dotted line) concentrations for change of influent Ac/N ratio in a chemostat enrichment culture. (A)**, Denitrification only. **(B)**, DNRA only. **(C)**, Denitrification and DNRA.

For a remarkably wide range of Ac/N ratios no nitrate or acetate could be detected in the effluent and both nitrate reduction processes co-existed. In case of one conversion, with one electron donor and one electron acceptor, e.g., when only denitrification or DNRA occurs, this dual limitation range is expected to be very narrow. This is shown in Figures 5A,B, where the effluent concentrations of acetate and nitrate are calculated assuming that either DNRA or denitrification occurs. Furthermore, these graphs (Figures 5A,B) show that the Ac/N ratio where both carbon and nitrogen limitation occur is strongly different for denitrification (Ac/N = 0.89) and DNRA (Ac/N = 1.36). This is due to the difference in the number of electrons transferred per unit of nitrate converted. This difference in stoichiometry between both processes prompts the double limitation for nitrate and acetate and co-occurrence of DNRA and denitrification over a broad range of Ac/N ratios.

It should be noted that if one process, DNRA or denitrification, would have a higher affinity for both acetate and nitrate, only one of these processes would occur over the full range of Ac/N values. Combined with the higher affinity for nitrate of DNRA and the higher affinity for acetate of denitrification, the difference in stoichiometry facilitates the broad range of Ac/N ratios where DNRA and denitrification coexist as adequately described by the model.

The model predicts that residual limiting substrate concentrations are higher in the dual substrate limitation range than in the single substrate limitation range (Figure 5C). This is the result of competition for both substrates, in which microorganisms do not manage to keep the substrate concentrations as low as for the case of a single substrate limitation. Under nitrate limiting conditions, the DNRA bacteria keep the nitrate well below a level attainable for denitrifying bacteria. At decreasing Ac/N ratios, the acetate concentration becomes limiting for the DNRA bacteria and nitrate starts to accumulate. At these increased nitrate concentrations and low acetate concentrations, the denitrifiers have a competitive edge due to their higher affinity for acetate. They can establish in the system up to the point where nitrate gets limited for the denitrifiers, and denitrification and DNRA can co-occur. As both organisms are pulling at both substrates in the transition phase, neither manages to outcompete the other, and they will coexist, albeit at higher residual substrate concentrations (Figure 5C).

Other factors, which are not considered in the model, possibly contributed to our observations of the Ac/N effect. The apparent higher affinity of denitrifiers for acetate might be a result of their higher competitiveness in comparison to the DNRA bacteria at increased redox potential imposed at nitrate excessive conditions. The low redox conditions at high Ac/N ratios might not only be influenced by the nitrate concentration but also facilitated by sulfate reducing bacteria, which are inevitably present in anoxic environments with sulfate and excess organic carbon present. Sulfide was not detected in the culture supernatant, but the small amounts produced could be directly consumed by the denitrifiers in autotrophic nitrate respiration. Micromolar amounts of sulfide are sufficient to strongly affect the redox potential of the system. Hence, the DNRA bacteria might depend on the sulfide producers to be able to persist in the reactor. A lower sulfide production in the reactor at lower Ac/N ratios in combination with the presence of nitrate would increase the redox potential. This increase might inhibit growth of the DNRA bacteria (Buresh and Patrick, 1981), and result in the domination of denitrifiers which are less sensitive to high redox.

The double substrate limitation described in this study deserves particular attention. For one, it underlines how hybrid the nitrate conversion is, with different conversion occurring simultaneously even in a simple system as a chemostat with one carbon- and energy source. Secondly, it differs from cases of double substrate limitations reported in literature. Most studies describing dual limitation of heterologous substrates, i.e., substrates that cannot be replaced by one another, study limitation of anabolic substrates in pure cultures (Egli, 1991; Zinn et al., 2004). Very few studies describe a dual limitation of heterologous catabolic substrates (Gottschal et al., 1979; van der Hoeven and Gottschal, 1989; Kovárová-Kovar and Egli, 1998). In the dual limitation of anabolic substrates, the dual limitation range is a result of the biomass flexibility to change composition (Zinn et al., 2004), which can be predicted by the biomass yields and is dependent on the dilution rate. In this study, the dual limitation is a result of the difference in stoichiometries, analogous to the yields in the anabolic substrate limitations, of denitrification and DNRA and not the biomass flexibility to change C/N content. As the C/N content of the biomass at different influent Ac/N ratios weres constant, we hypothesize that the observed dual limitation range is independent of the dilution rate. In the study of van der Hoeven and Gottschal (1989) on the dual limitation of the heterologous substrates glucose and oxygen in a mixture of two pure cultures, coexistence occurred as a result of the different susceptibilities of the cultures for substrate inhibition by oxygen. In our results, the occurrence of inhibition might be due to the millimolar excess of either acetate or nitrate, but appears unlikely since, at the pH used, the concentrations of candidates for toxicity, free acetate or nitrate, are very low.

The results described in this paper suggest that coexistence of DNRA and denitrification will occur in environments at a relative wide range of C/N supply ratios. Shifts in carbon or nitrate/nitrite loads may change the ratio of nitrate reduction products, but both processes will remain present. For example, in a wastewater treatment system with high organic carbon and nitrate in the influent (as in aquaculture and industrial wastewater) or nitrate presence in a recirculation stream which is mixed with the influent, DNRA bacteria can be active. Co-occurrence of denitrification and DNRA in artificial wastewater treatment wetlands and natural ecosystems, such as sediments, is often reported in literature (Mørkved et al., 2005; Giblin et al., 2013; Hardison et al., 2015). To which extent the Ac/N ratio dependent co-occurrence of DNRA and denitrification is influenced by the nature of the electron donor/carbon source in the system (Giblin et al., 2013; Plummer et al., 2015) and the oxidized nitrogen compound utilized (i.e., nitrite or nitrate), will be the topic of future studies. In addition, spatial heterogeneities in the environment could affect the co-occurrence (Verhagen and Laanbroek, 1991). Furthermore, one should not rule out the possibility that the organisms respond physiologically to the changing *in situ* concentrations of nitrate and acetate, by various metabolic mechanisms or regulatory effects (Gottschal and Thingstad, 1982; Gottschal, 1993).

### Microbial population

For the nitrate limiting conditions, the FISH and DGGE results indicated dominance of one organism. This organism was most closely related to *Geobacter luticola*. Although, *G. luticola* was shown to reduce nitrate to N_2_O (Viulu et al., 2013) and no DNRA activity was reported, the related organism found in this study is most likely performing the DNRA in our system.

The organism related to *G. lovleyi* strain SZ, which occurred after the shift of the Ac/N from 1.08 to 1.23 (Table 1), was assumed to perform the same conversions as the *G. luticola*-related strain in the reactor as it was 100% similar to the DNRA-performing dominant organism described previously by van den Berg et al. (2015). Additionally, the *G. luticola* related genotype had 97% sequence identity to *G. lovleyi*-related sequence and 96% to the *G. lovleyi* SZ. Hence, both the presumed DNRA-performing organisms in the studied system are closely related. Shifts between the two strains were seen earlier in this reactor (before the experiments of this study), while the conditions remained the same and the conversions were unaffected. Additionally, in the current experiments these organisms interchange in time (compare Table 1, Figure 3). Therefore, most likely the shift is not an effect of the different Ac/N ratio but rather a shift between two very similar organisms with very close affinities possibly affected by minor fluctuation in substrate supply (Gottschal, 1993).

Although, the population consists of >95% of the presumed DNRA organism under nitrate limitation, 15% of the nitrate was still reduced to dinitrogen gas. Either the DNRA bacteria are also capable of denitrification, like *Shewanella loihica* (Yoon et al., 2015), or the side population was denitrifying but with a relatively low growth yield. The low yield could be the results of other unknown factors such as the possible production of NO or N_2_O as byproducts by the DNRA bacteria (Costa et al., 1990; Torres et al., 2016) with the denitrifiers growing on these compounds rather than nitrate. Besides direct competition for the substrates nitrate and acetate, the bacteria can interact, which can be inhibitory or stimulatory (Gottschal and Thingstad, 1982), or they might have an effect on the regulation of enzymes or nutrient uptake system of one another (Gottschal, 1993). With the current experiments, the origin of the nitrogen formation was unidentifiable.

Under acetate limiting conditions, two dominant Betaproteobacterial genotypes were observed on the DGGE gel, while the *Geobacter* species had disappeared. One organism was most closely related to *Sulfurisoma sediminicola* and the other to *Variovorax boronicumulans. S. sediminicola* is a confirmed denitrifier (Kojima and Fukui, 2014), while *V. boronicumulans* is not (Miwa et al., 2008). However, closely related *Variovorax* species, for example *Variovorax paradoxus*, was shown to grow anaerobically with nitrate and acetate and its 16S RNA-gene is 98% similar to the genotype found in this study. The co-occurrence of the two denitrifiers under acetate limitation may be due to very close affinities and metabolic control of the two organisms. The sample for DNA extraction was collected relatively soon after the concentrations in the reactor became constant. A steady state situation in the concentrations in the chemostat does not necessarily mean a steady state in the population. If a bacterium has only a small net competitive advantage, it would need more time to outcompete others (Gottschal and Thingstad, 1982; Gottschal, 1993). As we did not run the steady state for more than five doubling times, we cannot disregard that possibility. Finally, it cannot be ruled out the two denitrifying organisms each perform a part of the denitrification pathway (Van de Pas-Schoonen et al., 2005).

When both nitrate and acetate were limiting, DNRA and denitrification coexisted and two organisms dominated in the microbial community, the *Geobacter* sp. and the *Azospira oryzae* (formerly *Dechlorosoma suillum*, Tan and Reinhold-Hurek, 2003)-related strain. *A. oryzae* was most likely responsible for the denitrification in the reactor. This is supported by the reported characteristics which indicated that *A. oryzae* was able of nitrate reduction with acetate whereby nitrate was completely reduced to nitrogen gas (Achenbach et al., 2001). As *A. oryzae* is most likely the *Betaproteobacterium* observed in FISH, it's abundance increased in the population with the increase in denitrification as a function of the Ac/N ratio in the feed (Tables 5, 6). For the steady state with dual limitation, but no ammonium production (Ac/N ratio of 0.93), the *A. oryzae* related organism was not observed and two other denitrifiers appeared to dominate. Both were related to *Sulfurisoma sediminicola*, as a closest cultured relative. Thus, the presumed DRNA bacteria dominant under nitrate limitation remained under dual limiting conditions, whereas the presumed denitrifying population changed as a result of the additional limiting factor.

At Ac/N ratio of 0.93, no ammonium production was observed, but the DNRA bacterium had remained, albeit at a low level, in the reactor, as was observed both with DGGE (band 3a, Figure 3) and FISH (data not shown). It could well be that all produced ammonia was so small that it was directly consumed and incorporated into the biomass of both organisms. However, the possibility remains that the *Geobacter*-related organism was performing mainly denitrification. When the feed was supplemented with an excess amount of ammonium, at Ac/N ratio of 0.93, the population did not change, not even after 20 doubling times. Although, the use of nitrate instead of ammonium for biomass results generally in a lower yield, no change in yields was measured within our accuracy. Many bacteria can use nitrate as a nitrogen source for growth (Gottschalk, 2012), while some obligatory depend on ammonia for growth, e.g., *Nitrolancetus hollandicus* (Sorokin et al., 2012). Probably the dominant bacteria in our system were able to use nitrate as an N-source, while retaining a competitive advantage.

### Conclusion

We showed a clear correlation between the Ac/N ratio and the prevalent dissimilatory nitrate reduction process in an open chemostat system using acetate as electron donor. Under nitrate limiting conditions DNRA was the dominant process while under acetate limiting conditions denitrification was dominant. Moreover, we demonstrated that for a substantial range of Ac/N supply ratios both substrates were limiting and denitrification and DNRA coexisted. The range of dual substrate limiting conditions can be explained as a result of both the stoichiometries of DNRA and denitrification and a higher affinity of the prevailing DNRA bacteria for nitrate and of the prevailing denitrifying bacteria for acetate. The presumed DNRA performing bacterium was most closely related to *Geobacter luticola* or *G. lovleyi* (*Deltaproteobacteria*). The presumed denitrifying population was dominated by 3 members of the *Betaproteobacteria*, belonging to *Azospira oryzae, Sulfurisoma sediminicola* and *Variovorax boronicumulans*. While the same DNRA bacteria were present under nitrate limitation as well as dual substrate limitation, the denitrifying community varied between acetate limited conditions and dual substrate limiting conditions. These insights into the mechanism of the competition between denitrification and DNRA helps improve our understanding of the N-cycle processes. This will be useful to predict the fate of nitrogen in different environments and contribute e.g., to the ability to predict eutrophication trajectories in aquatic environments or to evaluate potential impaired contribution of DNRA in wastewater treatment plants.

## Author contributions

EB wrote the manuscript and performed parts of lab work. MB performed most lab work. RK made the Excel model. All authors made substantial contributions to the experimental design, interpretation of the results and revisions of the manuscript.

## Funding

The investigation was supported by the BE-Basic Foundation, project number fp0702. ML was supported by the SIAM Gravitation Grant 024.002.002, the Netherlands Organization for Scientific Research.

### Conflict of interest statement

The authors declare that the research was conducted in the absence of any commercial or financial relationships that could be construed as a potential conflict of interest.

## References

[B1] Achenbach L. A. Michaelidou U. Bruce R. A. Fryman J. Coates J. D. (2001). Dechloromonas agitata gen. nov., sp. nov. and Dechlorosoma suillum gen. nov., sp. nov., two novel environmentally dominant (per) chlorate-reducing bacteria and their phylogenetic position. Int. J. Syst. Evol. Microbiol. 51, 527–533. 10.1099/00207713-51-2-527 11321099

[B2] Amann R. I. Binder B. J. Olson R. J. Chisholm S. W. Devereux R. Stahl D. A. (1990). Combination of 16S rRNA-targeted oligonucleotide probes with flow cytometry for analyzing mixed microbial populations. Appl. Environ. Microbiol. 56, 1919–1925. 2200342 10.1128/aem.56.6.1919-1925.1990 PMC184531

[B3] Brin L. D. Giblin A. E. Rich J. J. (2015). Effects of experimental warming and carbon addition on nitrate reduction and respiration in coastal sediments. Biogeochemistry 125, 81–95. 10.1007/s10533-015-0113-4

[B4] Buresh R. J. Patrick W. H. (1981). Nitrate reduction to ammonium an oranic nitrogen in an estaurine sediment. Soil Biol. Biochem. 13, 279–283. 10.1016/0038-0717(81)90063-8

[B5] Burgin A. J. Hamilton S. K. (2007). Have we overemphasized the role of denitrification in aquatic ecosystems? A review of nitrate removal pathways. Front. Ecol. Environ. 5, 89–96. 10.1890/1540-9295(2007)5[89:HWOTRO]2.0.CO;2

[B6] Costa C. Macedo A. Moura I. Moura J. J. G. Le Gall J. Berlier Y.. (1990). Regulation of the hexaheme nitrite/nitric oxide reductase of *Desulfovibrio desulfuricans, Wollinella succinogenes* and *Escherichia coli*. FEBS Lett. 276, 67–70. 10.1016/0014-5793(90)80508-G 2265715

[B7] Daims H. Brühl A. Amann R. Schleifer K.-H. Wagner M. (1999). The domain-specific probe EUB338 is insufficient for the detection of all *Bacteria*: development and evaluation of a more comprehensive probe set. Syst. Appl. Microbiol. 22, 434–444. 10.1016/S0723-2020(99)80053-8 10553296

[B8] Egli T. (1991). On multiple-nutient-limited growth of microorganisms. Antonie van Leeuwenhoek 60, 225–234. 10.1007/BF00430367 1687236

[B9] Giblin A. Tobias C. Song B. Weston N. Banta G. Rivera-Monroy V. (2013). The importance of Dissimilatory Nitrate Reduction to Ammonium (DNRA) in the nitrogen cycle of coastal ecosystems. Oceanography 26, 124–131. 10.5670/oceanog.2013.54

[B10] Gottschal J. C. (1985). Some reflections on microbial competitiveness among heterotrophic bacteria. Antonie van Leeuwenhoek 51, 473–494. 10.1007/BF00404494 3915196

[B11] Gottschal J. C. (1993). Growth kinetics and competition – some contemporary comments. Antonie van Leeuwenhoek 63, 299–313. 10.1007/BF00871225 8279826

[B12] Gottschal J. C. De Vries S. Kuenen J. G. (1979). Competition between the facultatively chemolithotrophic *Thiobacillus* A2, an obligately chemolithotrophic *Thiobacillus* and a heterotrophic spirillum for inorganic and organic substrates. Arch. Microbiol. 121, 241–249. 10.1007/BF00425062

[B13] Gottschal J. C. Thingstad T. F. (1982). Mathematical description of competition between two and three bacterial species under dual substrate limitation in the chemostat: a comparison with experimental data. Biotechnol. Bioeng. 24, 1403–1418. 10.1002/bit.260240612 18546432

[B14] Gottschalk G. (2012). Bacterial Metabolism. New York, NY: Springer.

[B15] Gujer W. Henze M. Mino T. Vanloosdrecht M. (1999). Activated Sludge Model No. 3. Water Sci. Technol. 39, 183–193. 10.1016/S0273-1223(98)00785-9

[B16] Hardison A. K. Algar C. K. Giblin A. E. Rich J. J. (2015). Influence of organic carbon and nitrate loading on partitioning between dissimilatory nitrate reduction to ammonium (DNRA) and N2 production. Geochim. Cosmochim. Acta 164, 146–160. 10.1016/j.gca.2015.04.049

[B17] Johnson K. Jiang Y. Kleerebezem R. Muyzer G. van Loosdrecht M. C. (2009). Enrichment of a mixed bacterial culture with a high polyhydroxyalkanoate storage capacity. Biomacromolecules 10, 670–676. 10.1021/bm8013796 19193058

[B18] Kelso B. Smith R. V. Laughlin R. J. Lennox S. D. (1997). Dissimilatory nitrate reduction in anaerobic sediments leading to river nitrite accumulation. Appl. Environ. Microbiol. 63, 4679–4685. 16535749 10.1128/aem.63.12.4679-4685.1997 PMC1389305

[B19] Kojima H. Fukui M. (2014). *Sulfurisoma sediminicola* gen. nov., sp. nov., a facultative autotroph isolated from a freshwater lake. Int. J. Syst. Evol. Microbiol. 64, 1587–1592. 10.1099/ijs.0.057281-0 24480906

[B20] Kovárová-Kovar K. Egli T. (1998). Growth kinetics of suspended microbial cells- from single-substrate-controlled growth to mixed-substrate kinetics. Microbiol. Mol. Biol. Rev. 62, 646–666. 9729604 10.1128/mmbr.62.3.646-666.1998 PMC98929

[B21] Kraft B. Strous M. Tegetmeyer H. E. (2011). Microbial nitrate respiration – Genes, enzymes and environmental distribution. J. Biotechnol. 155, 104–117. 10.1016/j.jbiotec.2010.12.025 21219945

[B22] Kraft B. Tegetmeyer H. E. Sharma R. Klotz M. G. Ferdelman T. G. Hettich R. L.. (2014). The environmental controls that govern the end product of bacterial nitrate respiration. Science 345, 676–679. 10.1126/science.1254070 25104387

[B23] Kuenen J. G. (2015). Continuous cultures (Chemostats), in Reference Module in Biomedical Sciences (Philadelphia, PA: Elsevier). 10.1016/B978-0-12-801238-3.02490-9 Available online at: http://www.sciencedirect.com/science/article/pii/B9780128012383024909

[B24] Loy A. Lehner A. Lee N. Adamczyk J. Meier H. Ernst J.. (2002). Oligonucleotide microarray for 16S rRNA gene-based detection of all recognized lineages of sulfate-reducing prokaryotes in the environment. Appl. Environ. Microbiol. 68, 5064–5081. 10.1128/AEM.68.10.5064-5081.2002 12324358 PMC126405

[B25] Manz W. Amann R. Ludwig W. Wagner M. Schleifer K.-H. (1992). Phylogenetic oligodeoxynucleotide probes for the major subclasses of Proteobacteria: problems and solutions. Syst. Appl. Microbiol. 15, 593–600. 10.1016/S0723-2020(11)80121-9

[B26] Miwa H. Ahmed I. Yoon J. Yokota A. Fujiwara T. (2008). *Variovorax boronicumulans* sp. nov., a boron-accumulating bacterium isolated from soil. Int. J. Syst. Evol. Microbiol. 58, 286–289. 10.1099/ijs.0.65315-0 18175723

[B27] Mørkved P. T. Søvik A. K. Kløve B. Bakken L. R. (2005). Removal of nitrogen in different wetland filter materials: use of stable nitrogen isotopes to determine factors controlling denitrification and DNRA. Water Sci. Technol. 51, 63–71. Available online at: http://wst.iwaponline.com/content/51/9/63.abstract 16042244

[B28] Neilson J. W. Jordan F. L. Maier R. M. (2013). Analysis of artifacts suggests DGGE should not be used for quantitative diversity analysis. J. Microbiol. Methods 92, 256–263. 10.1016/j.mimet.2012.12.021 23313091 PMC3957434

[B29] Plummer P. Tobias C. Cady D. (2015). Nitrogen reduction pathways in estuarine sediments: influences of organic carbon and sulfide. J. Geophys. Res. 120, 1958–1972. 10.1002/2015JG003057

[B30] Rütting T. Boeckx P. Müller C. Klemedtsson L. (2011). Assessment of the importance of dissimilatory nitrate reduction to ammonium for the terrestrial nitrogen cycle. Biogeosciences 8, 1779–1791. 10.5194/bg-8-1779-2011

[B31] Schäfer H. Muyzer G. (2001). Denaturing gradient gel electrophoresis in marine microbial ecology, in Methods in Microbiology, ed John H. P. (San Diego, CA: Academic Press), 425–468.

[B32] Scholten J. C. M. van Bodegom P. M. Vogelaar J. van Ittersum A. Hordijk K. Roelofsen W.. (2002). Effect of sulfate and nitrate on acetate conversion by anaerobic microorganisms in a freshwater sediment. FEMS Microbiol. Ecol. 42, 375–385. 10.1111/j.1574-6941.2002.tb01027.x 19709297

[B33] Silver W. L. Herman D. J. Firestone M. K. (2001). Dissimilatory nitrate reduction to ammonium in upland tropical forest soils. Ecology 82, 2410–2416. 10.1890/0012-9658(2001)082[2410:DNRTAI]2.0.CO;2

[B34] Sorokin D. Y. Lücker S. Vejmelkova D. Kostrikina N. A. Kleerebezem R. Rijpstra W. I.. (2012). Nitrification expanded: discovery, physiology and genomics of a nitrite-oxidizing bacterium from the phylum *Chloroflexi*. ISME J. 6, 2245–2256. 10.1038/ismej.2012.70 22763649 PMC3504966

[B35] Streminska M. A. Felgate H. Rowley G. Richardson D. J. Baggs E. M. (2012). Nitrous oxide production in soil isolates of nitrate-ammonifying bacteria. Environ. Microbiol. Rep. 4, 66–71. 10.1111/j.1758-2229.2011.00302.x 23757231

[B36] Tan Z. Reinhold-Hurek B. (2003). Dechlorosoma suillum Achenbach et al. 2001 is a later subjective synonym of *Azospira oryzae* Reinhold-Hurek and Hurek 2000. Int. J. Syst. Evol. Microbiol. 53, 1139–1142. 10.1099/ijs.0.02606-0 12892141

[B37] Tiedje J. M. Sextone A. J. Myrold D. D. Robinson J. A. (1982). Denitrification: ecological niches, competition and survival. Antonie van Leeuwenhoek 48, 569–583. 10.1007/BF00399542 6762848

[B38] Torres M. J. Simon J. Rowley G. Bedmar E. J. Richardson D. J. Gates A. J.. (2016). Nitrous oxide metabolism in nitrate-reducing bacteria: physiology and regulatory mechanisms. Adv. Microb. Physiol. 68, 353–432. 10.1016/bs.ampbs.2016.02.007 27134026

[B39] van den Berg E. M. van Dongen U. Abbas B. van Loosdrecht M. C. (2015). Enrichment of DNRA bacteria in a continuous culture. ISME J. 9, 2153–2161. 10.1038/ismej.2015.26 25909972 PMC4579468

[B40] Van de Pas-Schoonen K. T. Schalk-Otte S. Haaijer S. Schmid M. Op den Camp H. Strous M.. (2005). Complete conversion of nitrate into dinitrogen gas in co-cultures of denitrifying bacteria. Biochem. Soc. Trans. 33, 205–209. 10.1042/bst0330205 15667308

[B41] van der Hoeven H. S. Gottschal J. C. (1989). Growth of mixed cultures of *Actinomyces viscosus* and *Streptococcus mutans* under dual limitation of glucose and oxygen. FEMS Microbiol. Ecol. 62, 275–284. 10.1111/j.1574-6968.1989.tb03381.x

[B42] Verhagen F. J. M. Laanbroek H. J. (1991). Competition for ammonium between nitrifying and heterotrophic bacteria in dual energy-limited chemostats. Appl. Environ. Microbiol. 57, 3255–3263. 16348588 10.1128/aem.57.11.3255-3263.1991 PMC183957

[B43] Vishniac W. Santer M. (1957). The Thiobacilli. Microbiol. Mol. Biol. Rev. 21, 195–213. 13471458 10.1128/br.21.3.195-213.1957 PMC180898

[B44] Viulu S. Nakamura K. Okada Y. Saitou S. Takamizawa K. (2013). *Geobacter luticola* sp. nov., an Fe (III)-reducing bacterium isolated from lotus field mud. Int. J. Syst. Evol. Microbiol. 63, 442–448. 10.1099/ijs.0.039321-0 22493170

[B45] Yoon S. Cruz-García C. Sanford R. Ritalahti K. M. Löffler F. E. (2015). Denitrification versus respiratory ammonification: environmental controls of two competing dissimilatory ${\text{NO}}_{3}^{-}$/${\text{NO}}_{2}^{-}$ reduction pathways in *Shewanella loihica* strain PV-4. ISME J. 9, 1093–1104. 10.1038/ismej.2014.201 25350157 PMC4409154

[B46] Zinn M. Witholt B. Egli T. (2004). Dual nutrient limited growth: models, experimental observations, and applications. J. Biotechnol. 113, 263–279. 10.1016/j.jbiotec.2004.03.030 15380660

