# Supplementary material for: DNRA and Denitrification Coexist over a Broad Range of Acetate/N-NO3− Ratios, in a Chemostat Enrichment Culture
Source: Front Microbiol. 2016 Nov 24;7:1842. doi: 10.3389/fmicb.2016.01842 (PMC5121219; doi:10.3389/fmicb.2016.01842)
Supplement: Supplementary file 1 [file DataSheet1.DOCX]

Supplementary Material

Effect of COD/N ratio on the competition between DNRA and denitrification in a chemostat enrichment culture

Eveline M. van den Berg^1,*^, Marissa Boleij^1^, J. Gijs Kuenen^1^, Robbert Kleerebezem^1^ and Mark C. M. van Loosdrecht^1^

^1^Environmental Biotechnology group, Department of Biotechnology, Delft University of Technology, Delft, The Netherlands

*** Correspondence:** Eveline van den Berg: E.M.van den [Berg@tudelft.nl](mailto:Berg@tudelft.nl)

# Supplementary Figures


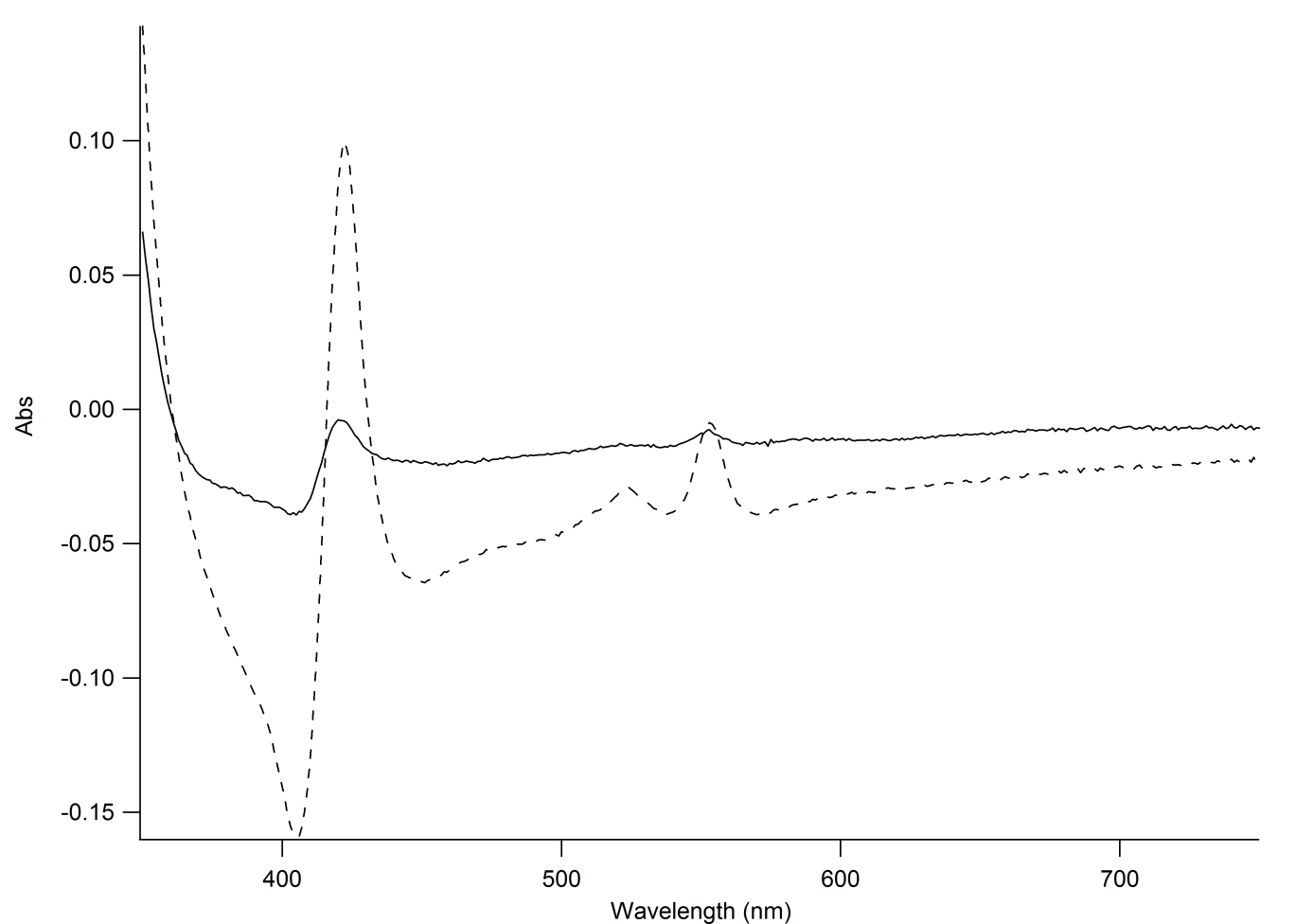


**Figure S1** Redox adsorption spectra of the DNRA culture (dotted line) and denitrifying culture (solid line) to illustrate the difference in heme content.


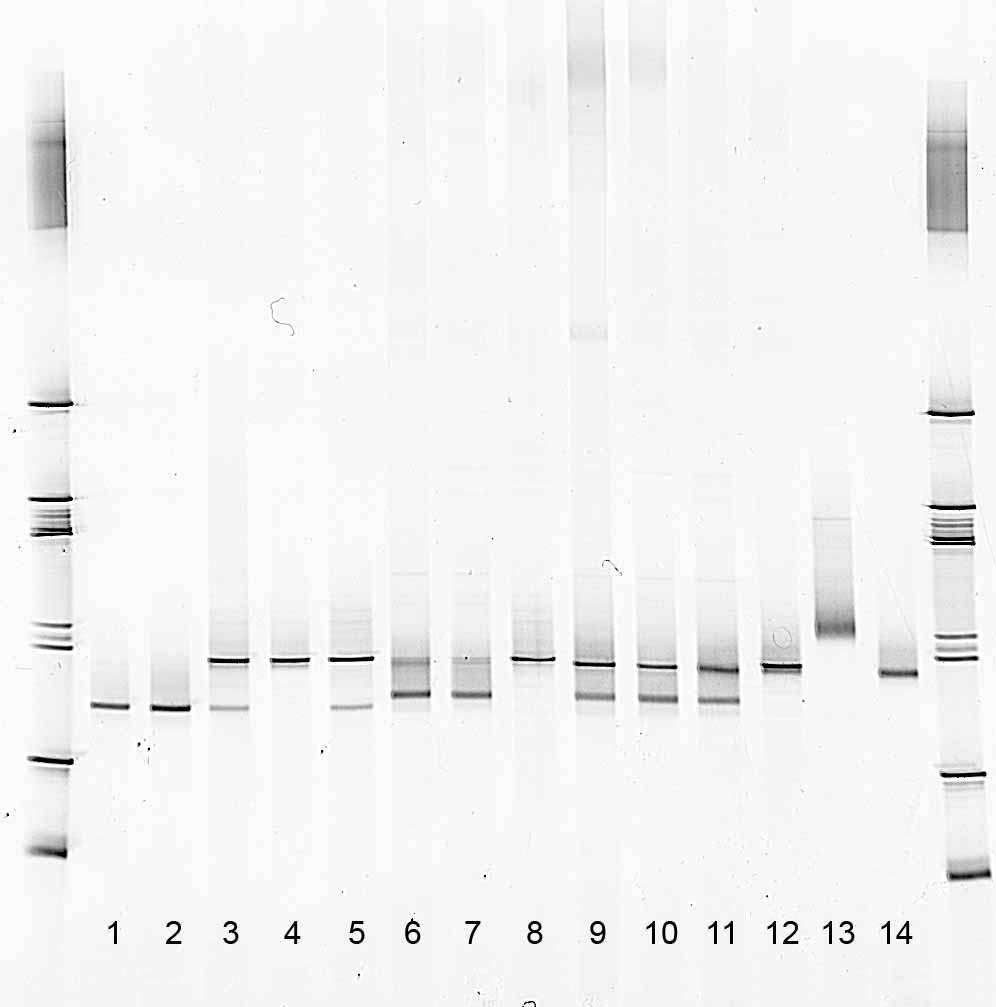


**Figure S2** Full DGGE gel picture. Lane 1-7 are lane A-G in figure 3. Lane 9 is lane H in figure 3. In lane 8 is a culture sample taken between the steady state of COD/N 5.3 and 3.0 g/gN (table 1). The sample in lane 10, like in lane 9, is a steady state sample of COD/N 3.0 g/gN, Lane 11 and 12 are two culture samples taken between the steady state of COD/N 4.3 to 5.3 g/gN (table 1). The samples in lane 13 and 14 belong to other research.
